# Altered expression of the TCR signaling related genes CD3 and FcεRIγ in patients with aplastic anemia

**DOI:** 10.1186/1756-8722-5-6

**Published:** 2012-03-08

**Authors:** Bo Li, Sichu Liu, Yuzhe Niu, Su Fang, Xiuli Wu, Zhi Yu, Shaohua Chen, Lijian Yang, Yangqiu Li

**Affiliations:** 1 Institute of Hematology, Medical College, Jinan University, Guangzhou 510632, China; 2 Key Laboratory for Regenerative Medicine of Ministry of Education, Jinan University, Guangzhou 510632, China

**Keywords:** Aplastic anemia, CD3γ, CD3δ, CD3ε, CD3ζ, FcεRIγ, Gene expression

## Abstract

**Background:**

Aplastic anemia (AA) is characterized by pancytopenia and bone marrow hypoplasia, which results from immune-mediated hematopoiesis suppression. Understanding the pathophysiology of the immune system, particularly T cells immunity, has led to improved AA treatment over the past decades. However, primary and secondary failure after immunosuppressive therapy is frequent. Thus, knowledge of the immune mechanisms leading to AA is crucial to fundamentally understand the disease.

**Findings:**

To elucidate the T cell receptor (TCR) signal transduction features in AA, the expression levels of CD3γ, δ, ε and ζ chain and FcεRIγ genes, which are involved in TCR signal transduction, and the negative correlation of the expression levels between the CD3ζ and FcεRIγ genes in T cells from peripheral blood mononuclear cells (PBMCs) were analyzed. Real-time RT-PCR using the SYBR Green method was used to detect the expression level of these genes in PBMCs from 18 patients with AA and 14 healthy individuals. The β2microglobulin gene (β2M) was used as an endogenous reference. The expression levels of the CD3γ, CD3δ, CD3ε and CD3ζ genes in patients with AA were significantly increased compared to a healthy control group, whereas the FcεRIγ gene expression level was significantly decreased in patients with AA in comparison with the healthy control group. Moreover, the negative correlation of the expression levels between the CD3ζ and FcεRIγ genes was lost.

**Conclusions:**

To our knowledge, this is the first report of the CD3γ, CD3δ, CD3ε, CD3ζ and FcεRIγ gene expression in patients with AA. The abnormally expressed TCR signaling related genes may relate to T cells dysfunction in AA.

## Introduction

Aplastic anemia (AA) is a potentially fatal bone marrow failure disorder that is characterized by pancytopenia and bone marrow hypoplasia. In most cases, AA behaves as an immune-mediated disease [1]. Immunosuppression is a key treatment strategy for patients who are not suitable bone marrow transplant (BMT) candidates due to age or the lack of a suitable donor. Immunosuppression with antithymocyte globulins and cyclosporine is effective for restoring blood cell production in the majority of patients [2,3]. The thought that T cells play a major role in the pathophysiology of AA became evident in the late 1970s with the finding that marrow and peripheral blood lymphocytes from patients with AA were able to suppress hematopoiesis in vitro. Subsequently, activated CD8^+ ^T cells were identified as the lymphocyte subset that inhibited hematopoiesis in AA patients [4]. Further studies have indicated that the mechanism includes Fas-induced apoptosis [5] and the release of several inhibitory cytokines including γ-interferon (IFN-γ), tumor necrosis factor-α (TNF-α) and transforming growth factor-β (TGF-β) [6-8]. Subsequent studies have demonstrated that oligoclonal expanded cytotoxic T cells target hematopoietic stem and progenitor cells [1]. All of these findings suggest that the molecular basis of the aberrant immune response and deficiencies in hematopoietic cells is T cells activation pathway dysregulation [9].

When T cells encounter antigens via the TCR, information about the quantity and quality of antigen engagement is relayed to the intracellular signal transduction machinery. The TCR lacks a significant intracellular domain. Instead, it associates with CD3 molecules, which contain intracellular signaling domains that couple the TCR/CD3 complex to downstream signaling machinery. The earliest TCR signaling events involve the transfer of information from the antigen-binding TCR subunit to the CD3 signaling subunits in the TCR/CD3 complex [10]. The Fc epsion receptor type Iγ (FcεRIγ) chain is a member of the CD3ζ chain protein family, and it is a component of the high-affinity IgE receptor FcεRI. There is evidence that the FcεRIγ chain can replace a functionally deficient CD3ζ chain and facilitate TCR/CD3 complex-mediated signaling [11,12].

Growing evidence has shown that AA is a type of autoimmune disease that involves a T cell attack against hematopoietic progenitor cells [13]. Oligoclonal T cells expansion was detected in autoimmune diseases such as systemic lupus erythematosus (SLE), rheumatoid arthritis and multiple sclerosis [14-17]. The TCR/CD3 signaling complex appears to be down-regulated by mutations/polymorphisms in autoimmune diseases, chronic inflammation and malignant tumors, which are thought to be related to T cells immunodeficiency [18,19].

Antigen-specific T cells play a central role in immune and inflammatory responses. An appropriate immune response by these cells depends on the careful regulation of their activation. However, little is known about the expression pattern of the CD3 complex and FcεRIγ genes in AA patients. We concluded that analysis of these genes in AA patients during their initial presentation may serve to identify the abnormal immune characteristics of AA.

## Methods

### Samples

The AA group consisted of 18 patients with newly diagnosed AA (12 males and 6 females; median age: 21 years, range: 8-73 years). Two of the cases were collected after immunosuppression treatment (ATG + CsA) at different time points (i.e., 1, 3 or 4 months). The information and clinical data of patients were described in Table 1 and 2. Fourteen healthy individuals (9 males and 5 females; median age: 24.5 years, range: 12-65 years) served as the control group. The AA diagnosis was established by bone marrow biopsy and peripheral blood counts. All of the procedures were performed according to the guidelines of the Medical Ethics committee of the health bureau of the Guangdong Province of China. Peripheral blood mononuclear cell (PBMC) isolation, RNA extraction and cDNA synthesis were performed according to the manufacturer's instructions.

**Table 1:** Clinical data of AA patient

Case number	Age (years)	Sex	Hb(g/L)	ANC (10^9^/L)	PLT (10^9^/L)
1	10	Female	60	0.1	69
2	17	Male	112	1.2	39
3	24	Male	40	0.3	42
4	12	Male	69	0.8	24
5	14	Female	55	0.4	29
6	28	Male	87	0.8	90
7	27	Male	67	0.3	45
8	19	Male	91	0.7	29
9	73	Male	59	1.1	94
10	15	Female	81	0.4	63
11	8	Female	65	0.4	12
12	19	Female	74	0.1	52
13	18	Male	71	0.1	45
14	48	Male	40	0.1	24
15	57	Male	83	0.3	30
16	31	Male	47	0.5	21
17	35	Female	63	0.1	20
18	26	Male	108	0.2	12

Note: Hb:haemoglobin; ANC: absolute neutrophil count PLT: platelet

**Table 2:** Clinical data of follow-up patients

Case number	Time	Hb (g/L)	ANC (10^9^/L)	PLT (10^9^/L)
1	Before treatment 4 months after treatment	60 66	0.1 0.6	69 45
2	Before treatment 1 months after treatment	112 119	1.2 1.1	39 43
	3 months after treatment	124	1.6	47

### Real-time relative quantitative PCR for CD3γδεζ and FcεRIγ genes

Real-time PCR using the SYBR Green I method was used to examine the CD3γ, δ, ε, ζ and FcεRIγ gene expression levels using cDNA obtained from the PBMCs of the 18 patients. The primer sequences and PCR conditions have been previously described [20]. Briefly, the PCR reactions were performed in a total volume of 25 μl containing approximately 1 μl cDNA, 0.5 μM of each primer pair, and 11.25 μl 2.5 × RealMasterMix (Tiangen, Beijing). After an initial denaturation at 95°C for 2 min, 45 cycles consisting of the following procedure was performed using an MJ Research DNA Engine Opticon 2 PCR cycler (BIO-RAD, Hercules, CA, USA): 15 s at 95°C; 1 min at 58.9°C for β2M and CD3γ, 60°C for CD3ζ and FcεRIγ, 60.8°C for CD3δ, and 62°C for CD3ε; and 1 s at 82°C for plate reading. The 2^(-ΔCT) ^method was used to analyze the results of the genes of interest relative to an internal control gene [21].

### Statistical analysis

An independent-samples *t *test was performed to compare the mean of each gene expression level between the patients with AA and the control groups. Pearson correlation and linear regression analyses were used to estimate the correlation between the CD3ζ and FcεRIγ gene expression levels from the different groups. A *P *< 0.05 was considered statistically significant.

## Results and discussion

The gene expression levels of CD3γ, CD3δ, CD3ε, CD3ζ and FcεRIγ in cDNA obtained from the PBMCs of 18 patients with AA before treatment and 14 healthy individuals were quantitatively assessed by real-time PCR using the SYBR Green I method.

**Figure 1:**
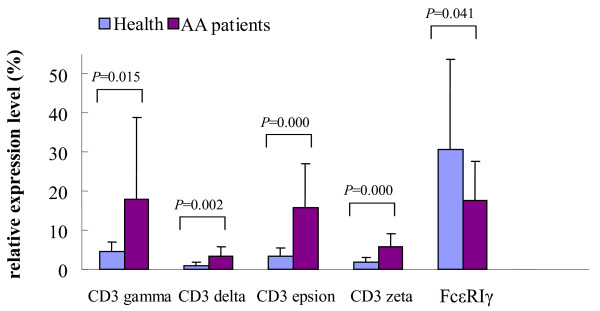
**The relative gene expression levels of CD3γ, CD3δ, CD3ε, CD3ζ and FcεRIγ in PBMCs from the AA and healthy groups**.

All five genes were detected in every sample. Significantly higher expression levels of CD3γ (17.84 ± 20.97, *p *= 0.015), CD3δ (3.24 ± 2.61, *p *= 0.002), CD3ε (15.73 ± 11.44, *p *= 0.000), and CD3ζ (5.65 ± 3.51, *p *= 0.000) were observed in patients with AA compared to the healthy controls (CD3γ (4.43 ± 2.67), CD3δ (1.02 ± 0.69), CD3ε (3.36 ± 2.09), and CD3ζ (1.83 ± 1.21) for the healthy controls; Figure 1). In contrast, significantly lower FcεRIγ expression levels (17.63 ± 9.95) were found in the AA group compared to the healthy group (30.55 ± 23.21, *p *= 0.041).

**Figure 2:**
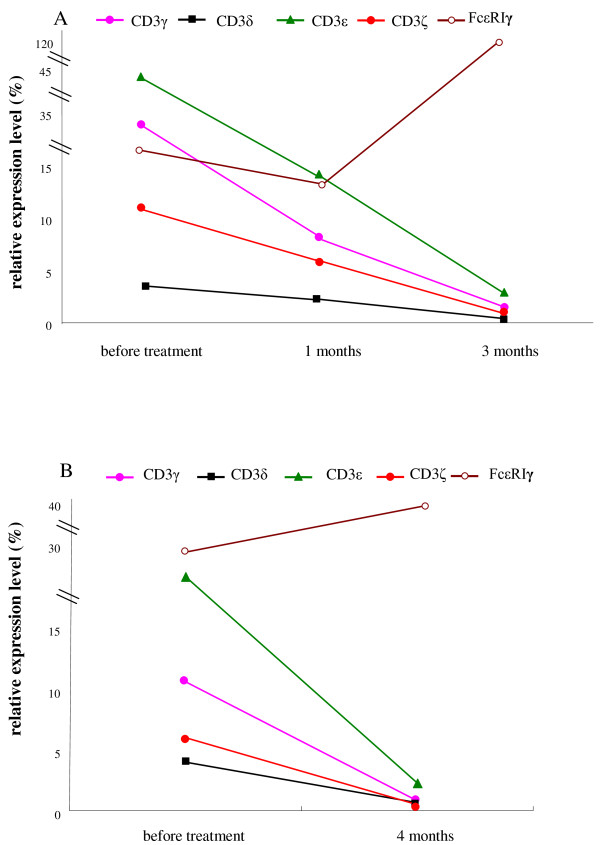
**The relative gene expression levels of CD3γ, CD3δ, CD3ε, CD3ζ and FcεRIγ in the PBMCs from 2 patients after treatment**.

The expression level of all five genes was compared in two patients with AA before and after treatment. The expression levels of the CD3γ, CD3δ, CD3ε and CD3ζ genes decreased, and the FcεRIγ expression level of increased after treatment (Figure 2A, B). The CD3γ expression level decreased from 34.03 to 1.31, the CD3δ expression level decreased from 3.36 to 0.52, the CD3ε expression level decreased from 45.06 to 2.96, the CD3ζ expression level decreased from 10.96 to 0.86, and the FcεRIγ expression level increased from 5.97 to 107.55 in the 3 months after treatment in 1 case (Figure 2A). The expression levels of CD3δ, CD3ε and CD3ζ returned to the normal range compared to the healthy group, whereas the CD3γ and FcεRIγ expression levels did not. In another case, the CD3γ expression level decreased from 10.57 to 0.82, the CD3δ expression level decreased from 3.95 to 0.63, the CD3ε expression level decreased from 19.14 to 2.23, the CD3ζ expression level decreased from 5.91 to 0.54, and the FcεRIγ expression level increased from 30.89 to 36.60 in the 4 months after treatment (Figure 2B). With the exception of the expression levels of CD3γ and CD3ζ, which remained out of the normal range, the expression levels of the other genes returned to normal.

**Figure 3:**
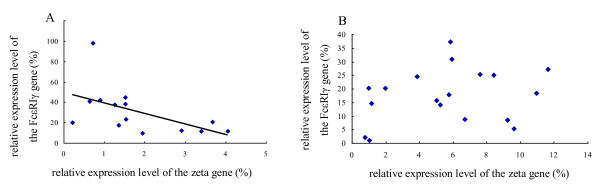
Correlation between the CD3ζ and FcεRIγ gene expression levels in PBMCs of the AA treatment and healthy groups (A: CD3ζ vs. FcεRIγ in the healthy group, the significant negative correlation is indicated; B: CD3ζ vs. FcεRIγ in the AA group)

A significant negative correlation between the CD3ζ and FcεRIγ gene expression levels was found in the healthy group (r = -0.535, *p *= 0.049; Figure 3A), while there was no significant correlation between the expression levels of the genes in the AA group (r = 0.252, *p *= 0. 313; Figure 3B).

The TCR has a short intracellular sequence that lacks signaling capacity, and the CD3 molecule contains immunoreceptor tyrosine-based activation motifs (ITAMs), which couple the TCR to the signal transduction mechanism. In mature T-lymphocytes, the TCR/CD3 complex can essentially trigger all adaptive immune responses [22,23]. TCR signal transduction is mediated by the ITAMs. A total of 10 ITAMs are distributed in the TCR/CD3 complex in two distinct signaling modules termed the TCRζζ and CD3γε/δε [24,25]. Although the TCRζζ ITAM plays a central role in TCR signal transmission, the CD3γε/δε ITAM module can provide normal TCR signal transmission in the absence of a TCRζζ ITAM motif [26]. To gain more insight into TCR signal transduction, which is important for T cells activation, we analyzed the expression level of all four CD3 genes (i.e., CD3γ, CD3δ, CD3ε and CD3ζ) and the CD3ζ-related FcεRIγ gene in PBMCs from patients with AA.

In contrast with recent studies that have revealed decreased CD3ζ chain protein and mRNA levels in most patients with AA, their results were derived from only five newly diagnosed patients with AA and patients (25 cases) in remission or relapse [27]. In this study, we analyzed the expression pattern of all of the CD3 genes, and the data indicated that not only the CD3ζ, but also the CD3γ, CD3δ and CD3ε expression levels were significantly increased in comparison with those in the healthy group. The results suggested that there may be a different T cells signaling pathway activation disorder in AA. Like many other cell surface receptors, TCR/CD3 complexes are constitutively internalized and recycled back to cell surface. This downmodulation of TCR/CD3 at the cell surface prevents sustained signalling in T-APC conjugates and modulates the responsiveness of T cells to further antigenic stimulation [28]. Up-regulation of CD3 gene expression in AA suggests that T cells may be in sustained signaling stimulation in the periphery that leads to inappropriate T cell activation.

Unlike the CD3ζ chain, which mediates signaling through ZAP-70, FcεRIγ mediates signaling by associating with the phosphorylated protein kinase Syk [12,29]. It has been reported that the Syk kinase is 100-fold more potent than Zap-70 [30], and it is preferentially recruited to the FcεRIγ [31]. Krishnan et al. have found a physiological switch through which primary resting CD4^+^T cells transduce signals to the classical TCR/CD3ε/CD3ζ/Zap-70 complex, and effector CD4^+^T cells express and transduce signals through an alternate TCR signaling complex that excludes CD3ζ/Zap-70 and contains CD3ε, the FcεRIγ subunit and the proximal Syk kinase [32]. It is interesting that this physiological switch in the TCR/CD3 signaling complex is not observed in CD8^+ ^T cells [33]. The TCR signaling alterations identified in our study suggest that the reduction in FcεRIγ expression in AA was unable to contribute to TCR signal transduction in a similar manner as the conserved functional ITAM motif.

In this study, we first analyzed the FcεRIγ gene expression level and its correlation with CD3ζ gene expression in patients with AA; the FcεRIγ expression level was down-regulated and did not correlate with the CD3ζ expression level. These findings are in contrast to the immune status in SLE [12], where a lower CD3ζ expression is associated with a higher FcεRIγ expression level. These data suggest that the different dysregulated T cells activation pathways involved in AA may be more complicated, and AA is unlikely to be a typical autoimmune disease T cells disorder. We were unable to follow up with the clinical outcome of all the patients who were selected for this study. We monitored the change in the CD3γ, CD3δ, CD3ε, CD3ζ and FcεRIγ gene expression levels in 2 patients with AA who were in remission. All of the gene expression patterns changed relative to the normal level. These data suggest that the expression of these genes is disease status dependent. However, a limited number of patients (2 cases) were examined in this follow-up study; thus, this hypothesis should be confirmed in future studies with larger sample sizes.

## Conclusions

To our knowledge, this study is the first attempt to provide a gene expression profile of the CD3γ, δ, ε, and ζ chains and the related FcεRIγ gene in PBMCs from patients with AA. Taken together, the increased T cells activation, along with the abnormally immunological molecules may be an AA feature. Abnormal T cells activation in AA creates a cellular imbalance between immunity and tolerance, leading to immune system-mediated bone marrow failure.

## Competing interests

The authors declare that they have no competing interests.

## Authors' contributions

YQL contributed to concept development and study design. BL, SHC, LJY performed the laboratory studies. SCL, YZN, SF, XLW and YZ were responsible for collection of clinical data. YQL and BL coordinated the study and helped drafting the manuscript. All authors read and approved the final manuscript.

## References

[B1] Young NS Scheinberg P Calado RT Aplastic anemia Curr Opin Hematol 2008 15 162 168 10.1097/MOH.0b013e3282fa7470 18391779 PMC3410534

[B2] Zhou F Ge L Yu Z Fang Y Kong F Clinical observations on intensive immunosuppressive therapy combined with umbilical cord blood support for the treatment of severe aplastic anemia J Hematol Oncol 2011 4 27 10.1186/1756-8722-4-27 21663651 PMC3128038

[B3] Sun ZM Liu HL Geng LQ Wang XB Yao W Liu X Ding KY Han YS Yang HZ Tang BL Tong J Zhu WB Wang ZY HLA-matched sibling transplantation with G-CSF mobilized PBSCs and BM decreases GVHD in adult patients with severe aplastic anemia J Hematol Oncol 2010 3 51 10.1186/1756-8722-3-51 21194460 PMC3023734

[B4] Zoumbos NC Gasco'n P Djeu JY Trost SR Young NS Circulating activated suppressor T lymphocytes in aplastic anemia N Engl J Med 1985 312 257 265 10.1056/NEJM198501313120501 2981406

[B5] Maciejewski JP Selleri C Sato T Anderson S Young NS Increased expression of Fas antigen on bone marrow CD34^+ ^cells of patients with aplastic anaemia Br J Haematol 1995 91 245 252 10.1111/j.1365-2141.1995.tb05277.x 7577642

[B6] Zoumbos NC Gascon P Djeu JY Young NS Interferon is a mediator of hematopoietic suppression in aplastic anemia in vitro and possibly in vivo Proc Natl Acad Sci USA 1985 82 188 192 10.1073/pnas.82.1.188 3918301 PMC396997

[B7] Sloand E Kim S Maciejewski JP Tisdale J Follmann D Young NS Intracellular interferon-gamma in circulating and marrow T cells detected by flow cytometry and the response to immunosuppressive therapy in patients with aplastic anemia Blood 2002 100 1185 1191 10.1182/blood-2002-01-0035 12149196

[B8] Dufour C Giacchino R Ghezzi P Tonelli R Ferretti E Pitto A Pistoia V Lanza T Svahn J Etanercept as a salvage treatment for refractory aplastic anemia Pediatr Blood Cancer 2009 52 522 525 10.1002/pbc.21886 19061218

[B9] Young NS Calado RT Scheinberg P Current concepts in the pathophysiology and treatment of aplastic anemia Blood 2006 108 2509 2519 10.1182/blood-2006-03-010777 16778145 PMC1895575

[B10] Call ME Pyrdol J Wucherpfenning KW Stoichiometry of T-cell receptor-CD3 complex and key intermediates assembled in the endoplasmic reticulum EMBO J 2004 23 2348 2357 10.1038/sj.emboj.7600245 15152191 PMC423287

[B11] Koyasu S D'Adamio L Arulanandam AR Abraham S Clayton LK Reinherz EL T cell receptor complexes containing Fc epsilon RI gamma homodimers in lieu of CD3 zeta and CD3 eta components: a novel isoform expressed on large granular lymphocytes J Exp Med 1992 175 203 209 10.1084/jem.175.1.203 1530959 PMC2119082

[B12] Enyedy EJ Nambiar MP Liossis SN Dennis G Kammer GM Tsokos GC Fc epsilon receptor type I gamma chain replaces the deficient T cell receptor zeta chain in T cells of patients with systemic lupus erythematosus Arthritis Rheum 2001 44 1114 1121 10.1002/1529-0131(200105)44:5<1114::AID-ANR192>3.0.CO;2-B 11352243

[B13] Kaito K Otsubo H Usui N Kobayashi M Th1/Th2 lymphocyte balance in patients with aplastic anemia Rinsho Byori 2004 52 569 573 15344555

[B14] Murata H Matsumura R Koyama A T cell receptor repertoire of T cells in the kidneys of patients with lupus nephritis Arthritis Rheum 2002 46 2141 2147 10.1002/art.10432 12209519

[B15] Kato T Kurokawa M Sasakawa H Masuko-Hongo K Matsui T Sekine T Tanaka C Yamamoto K Nishioka K Analysis of accumulated T cell clonotypes in patients with systemic lupus erythematosus Arthritis Rheum 2000 43 2712 2721 10.1002/1529-0131(200012)43:12<2712::AID-ANR11>3.0.CO;2-T 11145029

[B16] Thompson SD Murray KJ Grom AA Passo MH Choi E Glass DN Comparative sequence analysis of the human T cell receptor beta chain in juvenile rheumatoid arthritis and juvenile spondylarthropathies: evidence for antigenic selection of T cells in the synovium Arthritis Rheum 1998 41 482 497 10.1002/1529-0131(199803)41:3<482::AID-ART15>3.0.CO;2-G 9506577

[B17] Amemiya K Granger RP Dalakas MC Clonal restriction of T-cell receptor expression by infiltrating lymphocytes in inclusion body myositis persists over time. Studies in repeated muscle biopsies Brain 2000 123 2030 2039 10.1093/brain/123.10.2030 11004120

[B18] Ciszak L Pawlak E Kosmaczewska A Potoczek S Frydecka I Alterations in the expression of signal-transducing CD3 zeta chain in T cells from patients with chronic inflammatory/autoimmune diseases Arch Immunol Ther Exp (Warsz) 2007 55 373 386 10.1007/s00005-007-0042-6 18060371

[B19] Li Y Alterations in the expression pattern of TCR zeta chain in T cells from patients with hematological diseases Hematology 2008 13 267 275 10.1179/102453308X343482 18854088

[B20] Chen S Zha X Yang L Li B Zhong L Li Y Deficiency of CD3gamma, delta, epsilon and zeta expression in T-cells from AML patients Hematology 2011 16 31 36 10.1179/102453311X12902908411832 21269565

[B21] Livak KJ Schmittgen TD Analysis of relative gene expression data using real-time quantitative PCR and the 2(-Delta Delta C(T)) Method Methods 2001 25 402 408 10.1006/meth.2001.1262 11846609

[B22] Clevers H Alarcon B Wileman T Terhorst C The T-cell receptor/CD3 complex: A dynamic protein ensemble Annu Rev Immunol 1988 6 629 662 10.1146/annurev.iy.06.040188.003213 3289580

[B23] Call ME Wucherpfenning KW Molecular mechanisms for the assembly of the T cell receptor-CD3 complex Mol Immunol 2004 40 1295 1305 10.1016/j.molimm.2003.11.017 15072848 PMC4515969

[B24] Wegener AM Letourneur F Hoeveler A Brocker T Luton F Malissen B The T-cell receptor/CD3 complex is composed of at least two autonomous transduction modules Cell 1992 68 83 95 10.1016/0092-8674(92)90208-T 1531041

[B25] Malissen B An evolutionary and structural perspective on T-cell antigen receptor function Immunol Rev 2003 191 7 27 10.1034/j.1600-065X.2003.00016.x 12614348

[B26] Pitcher LA Mathis MA Young JA DeFord LM Purtic B Wulfing C van Oers NS The CD3 gamma epsilon/delta epsilon signaling module provides normal T cell functions in the absence of the TCR zeta immunoreceptor tyrosine-based activation motifs Eur J Immunol 2005 35 3643 3654 10.1002/eji.200535136 16259006

[B27] Solomou EE Wong S Visconte V Gibellini F Young NS Decreased TCR zeta-chain expression in T cells from patients with acquired aplastic anaemia Br J Haematol 2007 138 72 76 10.1111/j.1365-2141.2007.06627.x 17555449

[B28] Zanders ED Lamb JR Feldmann M Green N Beverley PC Tolerance of T-cell clones is associated with membrane antigen changes Nature 1983 303 625 627 10.1038/303625a0 6343888

[B29] Shiue L Zoller MJ Brugge JS Syk is activated by phosphotyrosine-containing peptides representing the tyrosine-based activation motifs of the high affinity receptor for IgE J Biol Chem 1995 270 10498 10502 10.1074/jbc.270.18.10498 7537732

[B30] Oliver JM Burg D Wilson BS McLaughlin JL Geahlen RL Inhibition of mast cell FcεRI-mediated signaling and effector function by the Syk-selective inhibitor, piceatannol J Biol Chem 1994 269 29697 29703 7961959

[B31] Taylor N Jahn T Smith S Lamkin T Uribe L Liu Y Differential activation of the tyrosine kinases ZAP-70 and Syk after FcgRI stimulation Blood 1997 89 388 396 9002939

[B32] Krishnan S Warke VG Nambiar MP Tsokos GC Farber DL The FcR gamma subunit and Syk kinase replace the CD3 zeta-chain and ZAP-70 kinase in the TCR signaling complex of human effector CD4 T cells J Immunol 2003 170 4189 4195 12682251 10.4049/jimmunol.170.8.4189

[B33] Kersh EN Kaech SM Onami TM Moran M Wherry EJ Miceli MC Ahmed R TCR signal transduction in antigen-specific memory CD8 T cells J Immunol 2003 170 5455 5454 12759421 10.4049/jimmunol.170.11.5455

